# 

**Published:** 1899-10-14

**Authors:** 


					THE HOSPITAL. "The standard of highest purity."?Lancet. October Uth, 1899.
CADBURY'S U few CADBURY'S
COCOA 1L ^ M Jt. COCOA
ABSOLUTELY PURE. NO DRUGS OR ALKALIES USED.
No. 681. JVol. XXVII. [fSSer!] Saturday,
Oct. 14th, 1899.
[-SuhscriptionTems,-] pRICE TWOPENCE.

				

